# Android and iPhone Mobile Apps for Psychosocial Wellness and Stress Management: Systematic Search in App Stores and Literature Review

**DOI:** 10.2196/17798

**Published:** 2020-05-22

**Authors:** Nancy Lau, Alison O'Daffer, Susannah Colt, Joyce P Yi-Frazier, Tonya M Palermo, Elizabeth McCauley, Abby R Rosenberg

**Affiliations:** 1 Department of Psychiatry and Behavioral Sciences University of Washington School of Medicine Seattle, WA United States; 2 Palliative Care and Resilience Lab Center for Clinical and Translational Research Seattle Children’s Research Institute Seattle, WA United States; 3 Department of Anesthesiology and Pain Medicine University of Washington School of Medicine Seattle, WA United States; 4 Center for Child Health, Behavior, and Development Seattle Children’s Research Institute Seattle, WA United States; 5 Cambia Palliative Care Center of Excellence University of Washington Seattle, WA United States; 6 Department of Pediatrics University of Washington School of Medicine Seattle, WA United States

**Keywords:** mHealth, mobile health, mental health

## Abstract

**Background:**

In an oversaturated market of publicly available mobile apps for psychosocial self-care and stress management, health care providers, patients, and consumers interested in mental health–related apps may wonder which, if any, are efficacious. Readily available metrics for consumers include user popularity and media buzz rather than scientific evidence.

**Objective:**

This systematic review aimed to (1) examine the breadth of therapeutic contents and features of psychosocial wellness and stress management apps available to self-help seekers for public download and (2) determine which of these apps have original research support.

**Methods:**

First, we conducted a systematic review of publicly available apps on the iPhone App Store (Apple Inc) and Android Google Play (Google LLC) platforms using conventional self-help-seeking search terms related to wellness and stress. The results were limited to English-language apps available for free download. In total, 2 reviewers independently evaluated all apps and discussed the findings to reach 100% consensus regarding inclusion. Second, a literature review was conducted on the included apps to identify supporting studies with original data collection.

**Results:**

We screened 3287 apps and found 1009 psychosocial wellness and stress management apps. Content varied widely. The most common evidence-based strategy was mindfulness-meditation, followed by positive psychology and goal setting. Most apps were intended to be used as self-help interventions, with only 1.09% (11/1009) involving an electronic therapist and 1.88% (19/1009) designed as a supplement to in-person psychotherapy. Only 4.66% (47/1009) of apps targeted individuals with psychological disorders, and less than 1% of apps (6/1009, 0.59%) targeted individuals with other chronic illnesses. Approximately 2% (21/1009, 2.08%) were supported by original research publications, with a total of 25 efficacy studies and 10 feasibility studies. The *Headspace* mindfulness app had the most evidence, including 8 efficacy studies. Most other scientifically backed apps were supported by a single feasibility or efficacy study.

**Conclusions:**

Only 2.08% (21/1009) of publicly available psychosocial wellness and stress management mobile apps discoverable to self-help seekers have published, peer-reviewed evidence of feasibility and/or efficacy. Clinicians and investigators may use these findings to help patients and families navigate the volume of emerging digital health interventions for stress management and wellness.

## Introduction

### Background

Within the past decade, smartphones have become ubiquitous in personal, social, and work life [1], irrespective of gender, race and ethnicity, and socioeconomic status [2]. Overall, 75% of Americans own a smartphone, and 83% of them never leave home without it [1,3]. On average, a person checks his or her phone 150 times per day [4]. Owing to the pervasiveness of smartphones in modern day culture, technological innovations may be leveraged to disseminate *in the moment* behavioral change interventions designed to promote healthy behaviors [5]. There is a robust market for health apps, with 325,000 available for download as of 2017 and a growth rate of 25% year to year [6]. More than half of mobile phone users have downloaded a health-related mobile app, and the pace of development of evidence-based apps tested in research settings has lagged far behind than that of the commercial sector [7-9].

In particular, mobile health (mHealth) apps focused on promoting emotional health and adaptive coping have become increasingly popular. Mental health symptoms such as anxiety and stress are prevalent and disruptive. Overall, 75% of adults in the United States report significant stress, and 19% have mental health disorders [10]. Anxiety disorders impact up to 30% of individuals worldwide, leading to severe societal and economic burden [11]. Work-related stress alone costs the US economy US $402 billion [12]. Disseminating psychosocial interventions via mHealth technologies confers the advantage of universal accessibility regardless of geographic and economic restrictions [1,13]. According to the US National Comorbidity Survey (a nationally representative large-scale mental health study), common barriers to seeking mental health care include financial constraints, stigma, and a desire for self-management of symptoms [14]. In other studies, most individuals reported interest in using a mobile app for self-management of anxiety, stress, and depression if services were available for free [8,15]. In total, two recent meta-analyses of randomized controlled trials (RCTs) showed that mHealth interventions for anxiety and depression showed small positive effects when compared with an active control condition [11,16].

Despite the high demand and potential advantages of these apps, there is a lack of quality control standards or readily accessible information to consumers on whether or which apps work in an oversaturated market. Thus, leveraging mHealth technologies brings both benefits and new challenges. In efforts to review publicly available apps using a direct-to-consumer approach, recent mHealth reviews have used a search strategy that involves entering key terms directly into the search engines of mobile app platforms [17-20]. In a review of iOS App Store mobile apps on Apple devices (Apple Inc), researchers identified 60 mobile apps that delivered at least one evidence-based stress management strategy (eg, mindfulness, progressive muscle relaxation, and biofeedback) [19]. A recent review of apps for depression, anxiety, posttraumatic stress disorder (PTSD), and alcohol use found that *evidence-based mobile apps* (ie, apps tested via formal research methods and published in the scientific literature) are often unavailable for download to the general public; in addition, apps available to consumers on commercial platforms are highly variable with regard to the inclusion of *evidence-based content* (ie, content derived from empirically supported therapeutic approaches) [20].

### Objectives

With an overabundance of publicly available apps for stress management and psychosocial self-care, consumers may struggle with a paradox of choice, regardless of whether they are providers seeking to make app recommendations, patients seeking additional mental health support, or app-savvy digital natives interested in self-help. Readily available metrics are app visibility because of ranked lists, user popularity, media buzz, and user satisfaction ratings. When an app purports to be based in science, its *scientific backing* may not reach the classical standards of research rigor. It remains unclear whether popular apps that consumers gravitate toward *work*. In this study, we broadly reviewed popular apps available to all manner of consumers (ie, the general public, patients, and providers) for free download and their treatment content, user ratings, costs, and the evidence base in support of them. We presented findings from a review of 1009 publicly available mobile apps on Apple Store and Google Play (Google LLC) platforms using common self-help-seeking search terms for psychosocial wellness and stress management. Our review spanned Apple and Android devices that together represent 99% of the smartphone user market; 54.4% of US smartphone owners use Android devices and 44.3% use Apple devices [21]. After systematically searching both mobile app platforms (step 1), we supplemented this direct-to-consumer approach by conducting a literature review of the apps identified (step 2).

Our research questions were as follows: (1) What are the active therapeutic components and features of publicly available psychosocial wellness, coping, and stress management mobile apps? (2) Do any of these mobile apps have evidence in support of their feasibility/acceptability or efficacy in the published scientific literature? We hypothesized that the majority of consumer apps identified would not contain evidence-based therapeutic strategies, and even fewer would have published research supporting the apps themselves. We translated research findings to clinical practice by describing the breadth of popular wellness apps for stress management and by identifying the few apps with scientific backing.

## Methods

### Searching the Apple Store and Google Play: Step 1

We systematically identified and evaluated apps using a modified version of the Preferred Reporting Items for Systematic Reviews and Meta-Analyses guidelines [22]; adjustments were made because of the differing methodology of directly searching app store platforms. Our search strategy protocol is available in Multimedia Appendix 1.

We searched the mobile app platforms App Store iOS (Apple Inc) and Google Play in September 2018. Inclusion criteria were (1) a focus on stress management and/or psychosocial wellness, (2) available in English, and (3) free for download (including those with free downloads for *basic* subscriptions, with additional fees for extra features).

Specifically, we first created a list of conventional self-help-seeking search terms from mental health and positive psychology background literature [23-31]; we refined the list in discussions among our interdisciplinary research team, which includes intervention science researchers, health services researchers, physicians, social workers, and psychologists. Then, we entered into Apple Store and Google Play search engines the 14 conventional self-help-seeking search terms agreed upon by our team: stress, resilience, goal setting, relaxation, mindfulness, mood, coping, gratitude, optimism, hope, happiness, sadness, self-compassion, and self-care. We noted that the app results were displayed on both search engines in the order of popularity, based on proprietary algorithms. Hence, we screened the first 100 apps populated for each search term. Indeed, research suggests that smartphone users limit their searches to the first page of results (which contains 10 apps) [32], so screening the first 100 apps was deemed sufficient. In addition, we screened the popularity lists in *health and fitness* and *kids and family* categories for apps that met the inclusion criteria (these popularity lists are displayed on Apple and Google Play platforms). Two authors (NL and AO) independently reviewed all apps for inclusion and discussed the findings to reach 100% consensus. Duplicates were removed.

### Data Extraction Procedures

For each app that met the inclusion criteria, 2 authors (NL and AO) extracted the following App Store iOS (Apple Inc) and Google Play product page data from November 2018 to February 2019 and discussed findings to reach 100% consensus: intervention and didactic content, target audience, and whether there were in-app paid features. In creating our database of intervention and didactic content, we used an all-inclusive approach to delineate content categories. For example, if an app description included meditation, mood tracking, artificial intelligence, and chat forums, we created unique content categories for each. Our goal was to provide a comprehensive representation of all intervention and didactic content as described on product pages by the app developers. To do so, we iteratively coded apps in sets of 50 and expanded the number of content categories as needed until there were no new content categories that arose. This resulted in the final version of the database, which contained 31 unique intervention and didactic content categories. During consensus conversations in March 2019 to April 2019, our process was to re-review App Store iOS (Apple Inc) and Google Play product page data to resolve discrepancies.

### Literature Review: Step 2

After all mobile apps were identified in step 1, 2 authors (AO and SC) conducted a literature review via Google Scholar, MEDLINE, and PsycINFO databases using the search terms *[app name]* AND *smartphone* from April 2019 to June 2019 to identify peer-reviewed papers supporting each of the identified apps. Furthermore, 2 authors (NL and AO) retrieved and independently reviewed the full text of all eligible studies to extract relevant feasibility and efficacy outcomes. We included research papers published in peer-reviewed journals and in English and included qualitative and/or quantitative studies with original data collection. We excluded conference presentations, editorials, commentaries, and study protocols. In consultation with a medical librarian, we chose to exclude nonpeer-reviewed scholarly works before publication in a peer-reviewed journal because the information included in conference abstracts lacks the rigor and external validity inherent in peer review. From the 33 included papers, we retrieved the following information: participants, sample size, study type, treatment conditions, and outcomes reported. Two authors (NL and AO) independently assessed study quality using the Cochrane collaboration’s tool for assessing the risk of bias [33], evaluating random sequence generation (selection bias), allocation concealment (selection bias), blinding of participants and personnel (performance bias), blinding of outcome assessment (detection bias), incomplete outcome data (attrition bias), selective reporting (reporting bias), and other bias. We coded each category as low, high, or unclear risk of bias according to established standards. We resolved minor discrepancies in coding by referring to the journal papers themselves.

## Results

### Searching the Apple Store and Google Play: Step 1

**Figure 1:**
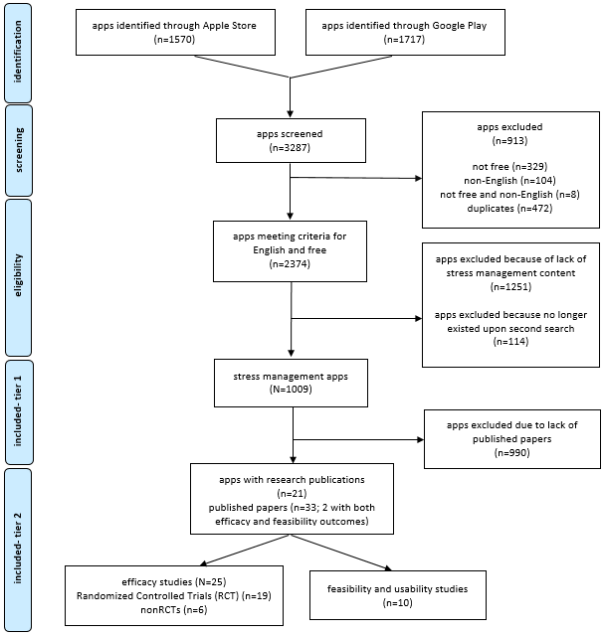
Preferred reporting items for systematic reviews and meta-analyses diagram.

We screened a total of 3287 apps (Figure 1). Of 3287 apps, 913 (27.78%) were excluded after the initial screening process because they were not available for free download, not in English, or were duplicates. Of the remaining 2374 apps, 1251 (52.70%) were excluded because they did not contain psychosocial wellness or stress management content (eg, health and fitness apps for exercise, nutrition, and weight loss). Of the remaining 1123 apps, 114 (10.15% that we initially found on the Apple Store or Google Play no longer existed 3 months later when authors attempted to refer to the original source for consensus conversations in March 2019 to April 2019. We ultimately included 1009 apps in this review.

#### Characteristics of All Included Apps (N=1009)

**Figure 2:**
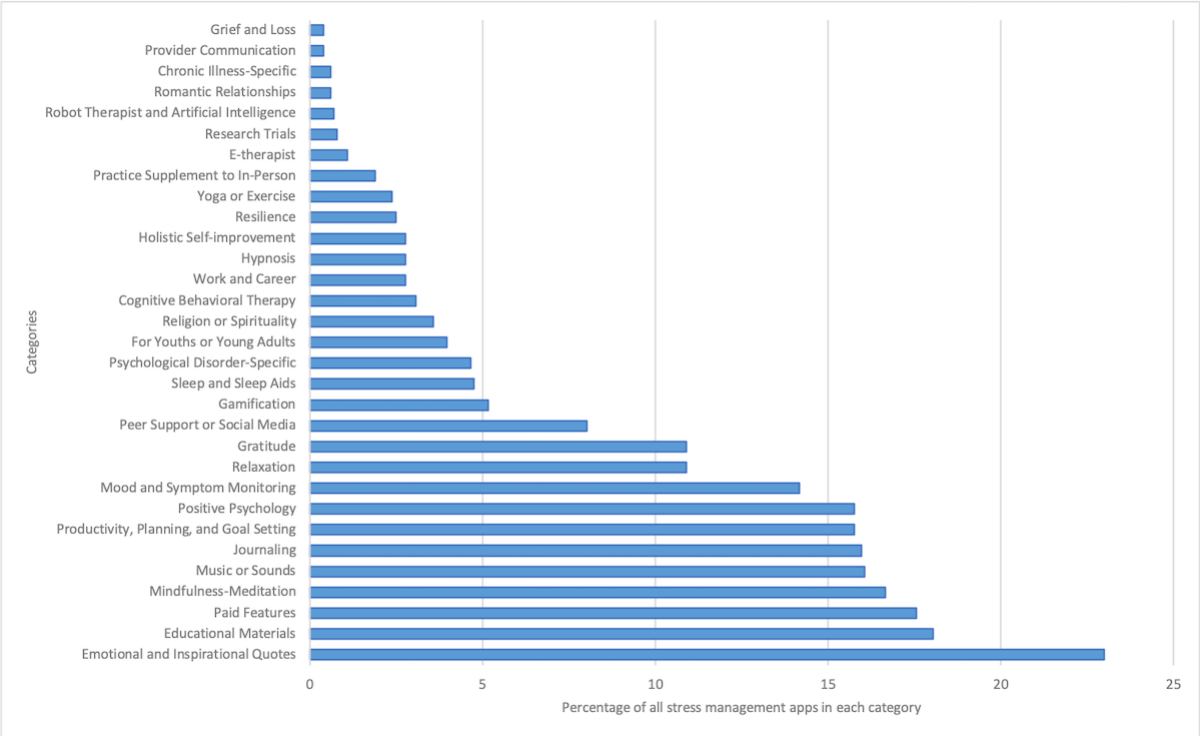
Intervention and didactic content for all stress management apps (N=1009). Content categories were assigned based on descriptions by the app developer. Categories were not mutually exclusive, and a single app could be represented across one or more. E-therapist: electronic therapist.

For the pooled 1009 apps, we found 31 unique intervention and didactic content categories (eg, cognitive behavioral therapy, mindfulness-meditation, and journaling; Figure 2). Emotional-inspirational quotes were the most common app component, included in 22.99% (232/1009) of apps. Other common components included in 15% of apps or more were goal setting, positive psychology, journaling, music, mindfulness-meditation, and educational materials. Only 4.66% (47/1009) of apps were designed specifically for psychological disorders, less than 1% of apps were designed for chronic illnesses (6/1009, 0.59%), and 3.96% (40/1009) of apps were designed for youths and/or young adults. Most apps were intended to be used as self-help interventions, with only 1.09% (11/1009) involving an electronic therapist (e-therapist) and 1.88% (19/1009) designed as a supplement to in-person psychotherapy.

### Literature Review: Step 2

#### Characteristics of Subset of Apps With Research Support (n=21)

**Figure 3:**
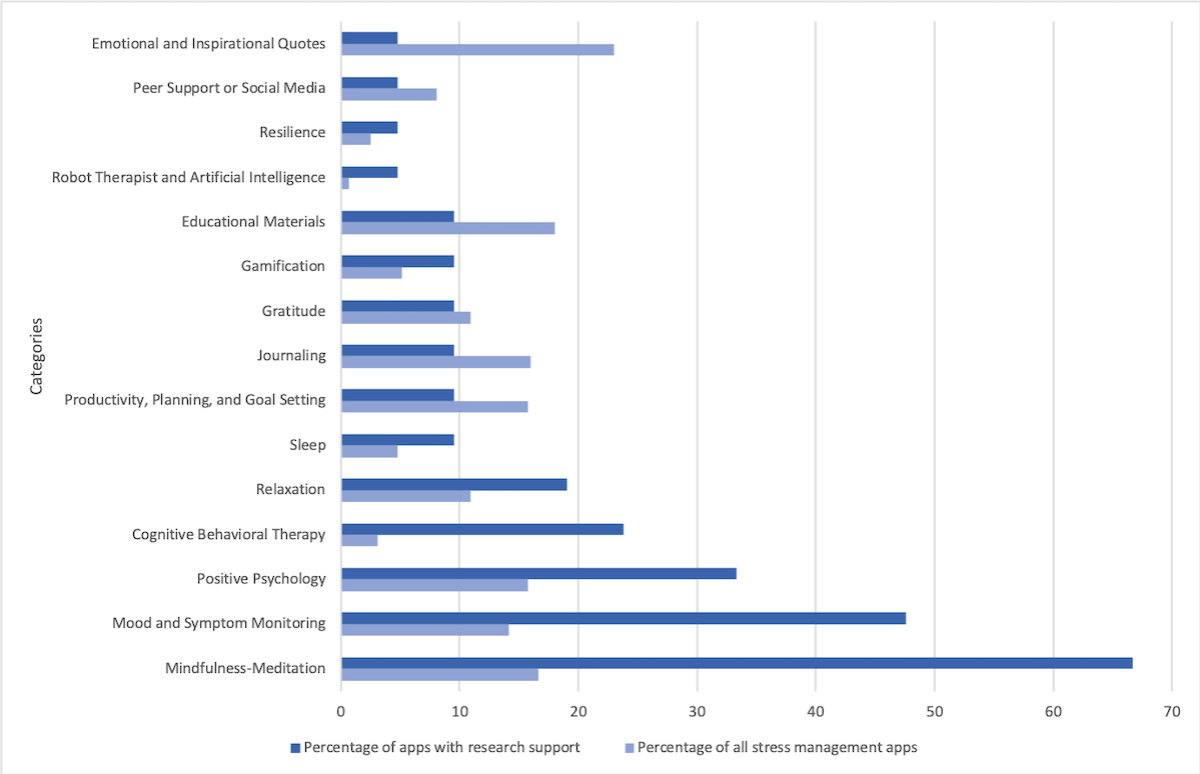
Intervention and didactic content for research-supported apps (n=21) vs all stress management apps (N=1009). Content categories were assigned based on descriptions by the app developer. Categories were not mutually exclusive, and a single app could be represented across one or more.

We found supporting original research publications for 2.08% of apps (21/1009 identified). For this subset of apps, the most common therapeutic component was mindfulness-meditation, an evidence-based treatment strategy that was incorporated into 67% (14/21) of apps with published research followed closely by mood and symptom monitoring. All other common app features (included in ≥15% of apps) were also evidence-based treatment strategies: cognitive behavioral therapy, positive psychology, and relaxation (Multimedia Appendix 2 and Figure 3). For each app, the average user satisfaction ratings and the number of user ratings varied widely (Multimedia Appendix 3).

#### Peer Review Publications

A total of 33 peer-reviewed papers supported the 21 apps; 23 of these papers were efficacy studies [34-58], 8 were feasibility or usability studies, and 2 were combined efficacy and feasibility studies [39,59]. For each of the 21 apps with research support, the number of associated peer-reviewed publications ranged from 1 to 8 (*Headspace* [46,48-54]; Multimedia Appendix 3). The majority of apps (16/21, 76%) only had 1 publication [34,35,37,38,43,44,46,55,56,58-62]. *10% Happier*, *Calm*, and *Headspace* were the only research-supported apps we found on ranked *health and fitness* popularity lists.

Of the 25 efficacy studies, 19 were RCTs published between 2015 and 2019 [34-37,40-42,44-46,48,49,51-56,58,59], with 6 of the 19 trials testing *Headspace* [46,48-54]. The majority of studies used samples of convenience, that is, college students [34,35,40,46,51,57] or users who had already downloaded the app [37,39,52,55,56,58]. Sample sizes for efficacy studies ranged from 19 [48] to 153,834 [39]. Treatment duration ranged from a single session of self-directed app use [39] to 6 months [38]. All 16 apps with peer-reviewed publications that reported app efficacy showed some evidence of improving psychosocial outcomes over time (Multimedia Appendix 4). Studies collected varying outcome measures, ranging from unstructured self-directed app use [41-45,50,62] to providing a sequential program of set frequency and duration [49,53]. In a subset of studies where effect sizes were reported; treatment effects ranged from small to large (Multimedia Appendix 4) [41,50,53,58]. Of the 10 apps with feasibility or usability studies, the majority (8/10 apps) reported that users found the apps to be enjoyable, accessible, and acceptable (Multimedia Appendix 5) [39,59-67]. Sample sizes for feasibility and usability studies ranged from 1 [60] to 1255 [64].

#### Risk of Bias Assessment

**Figure 4:**
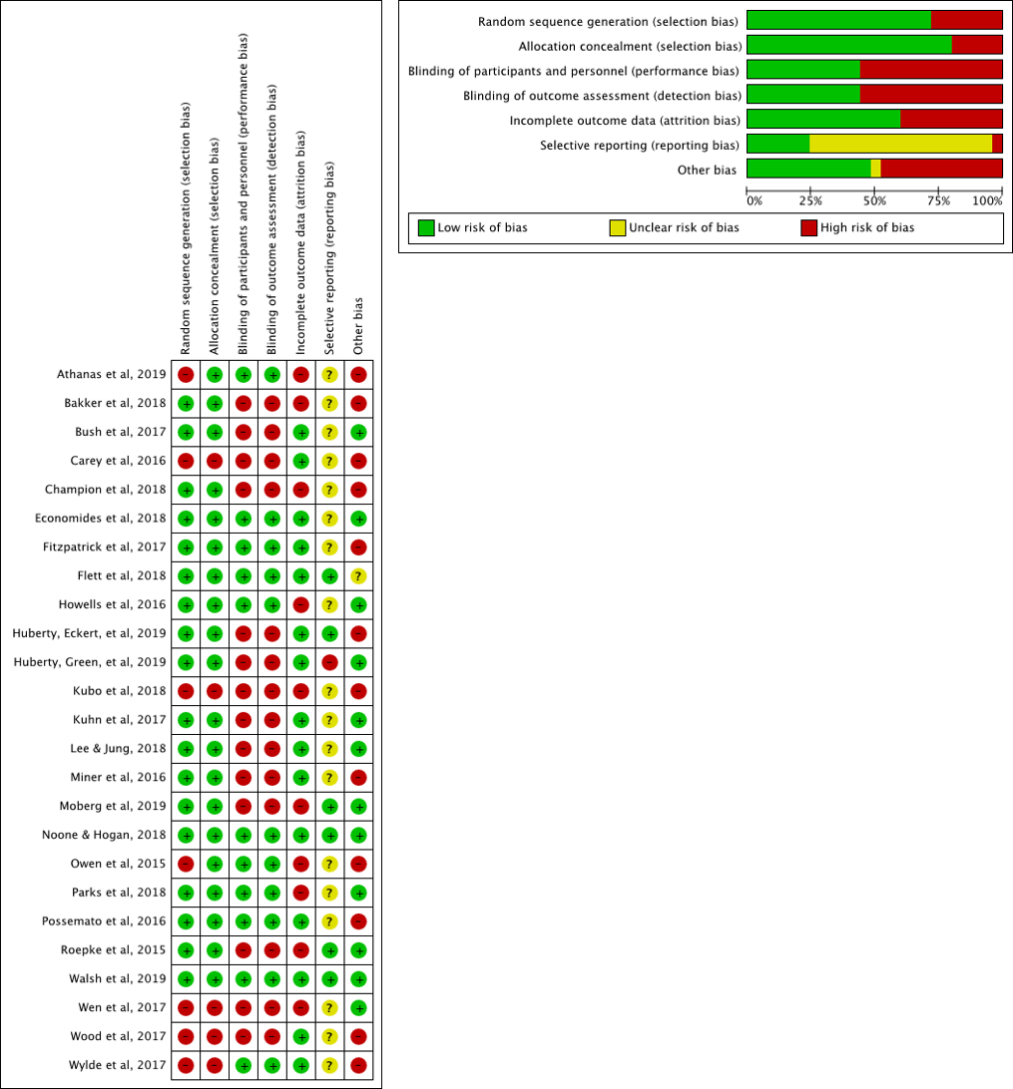
Summary of risk of bias.

The risk of bias was evaluated for all 25 efficacy studies (Figure 4). Of the 19 RCTs, 18 reported random sequence generation and allocation concealment. For the blinding of participants and personnel domain and the outcome assessment domain, studies were roughly split in half between high and low risk; high-risk studies consisted of study designs with no control group, a waitlist control group, or an educational handout control group. For selective reporting bias, 6 were considered low risk, 1 high risk, and 18 were unclear. For other biases, 12 were considered low risk, 12 were high risk, and 1 was unclear.

## Discussion

### Principal Findings

To our knowledge, this is the first review of psychosocial wellness and stress management apps using a multilevel search strategy of mobile app search engines (ie, what self-help consumers would find) followed by a literature review (ie, what scientists would find). We aimed to explore treatment features and components commonly folded into mainstream apps and whether and how these differed from those of apps tested in research and clinical trials. In addition, we summarized the existing literature on all identified apps.

We identified 1009 stress management and psychosocial wellness apps on the Apple Store and Google Play. App contents and features were varied and eclectic, ranging from journaling to hypnosis. Of the 5 most common treatment features and components for all apps, only 1 was evidence based (mindfulness-meditation). Unsurprisingly, the subset of apps with research publications was much more cohesive; the 5 most common therapeutic components identified in this group of apps were all evidence based. We found that 2.08% (21/1009) of apps identified for inclusion had supporting research, and the majority of apps had only 1 research publication. All the published efficacy studies demonstrated some evidence that the app *works*, although effect sizes were rarely reported. However, the *file-drawer problem* [68] in academic research (ie, studies with null findings are less likely to be published) and contrary business incentives (ie, publishing null findings does not make for a marketable app) may contribute to potential publication biases that highlight positive effects. The majority of published feasibility studies demonstrated some evidence of user satisfaction and acceptability. Our research expands on findings from classic methodology systematic reviews of smartphone-based anxiety and depression interventions that have found small to moderate positive effects [11,16].

Although there is a surfeit of mobile apps available for free download, only a small fraction of these have been tested in research settings. Even for the few published studies, the state of the science of mHealth for stress management and emotional wellness is in its nascent stages. Roughly half of the efficacy studies included in our review were either non-RCTs or RCTs without an active treatment comparison condition (ie, waitlist control). Approximately, one-fourth of all included studies were feasibility studies that did not measure efficacy outcomes. The majority of studies were not powered for analysis and, therefore, did not designate a priori primary vs secondary outcomes. Thus, the rapidly growing consumer market of mHealth for mental health is facing a similar *research-practice gap* to that of traditional face-to-face interventions: an overwhelming majority of self-help seekers may not be receiving evidence-based care [20]. This is not to discount the fact that businesses may have internally rigorous research and development processes outside of publications in scientific journals that we are unable to track or evaluate in a systematic way.

The World Health Organization (2019) recently released a guideline on digital health interventions for strengthening health systems based on an assessment of the benefits, harms, acceptability, feasibility, resource use, and equity considerations. The guideline’s primary objective is the adoption of evidence-based interventions [69]. The European Commission provides complementary guidelines, including an assessment of data protection and privacy, safety, scientific content, and effectiveness [70]. In practice, mHealth interventions developed and tested in formal research settings for research purposes are rarely made available to the general public [71].

Potential future directions for traversing the research-practice divide are for academic researchers to partner with health technology companies and businesses to develop and test publicly available apps [72]. Such collaborations would improve the rigor of app development and continuous refinement by applying quality control standards to an unregulated market while capitalizing on the strengths of the commercial sector in financial and personnel resources, innovation, marketing, and motivational factors for user engagement. The app development process in the commercial sector adheres to a *user-centered design* framework, which engages end users as part of an iterative design process to better understand facilitators and barriers to sustainability and use [73]. This is a crucial model to apply in mHealth research because of historically low user adherence and retention rates. In addition, it is important to bolster the representation of other known evidence-based strategies in app components and features such as cognitive behavioral therapy, which was only represented in 3.07% (31/1009) apps identified. This was consistent with a previous review of popular anxiety and depression mobile apps that found evidence-based treatment strategies were poorly represented [74]. Finally, there is a need for comprehensive, consolidated, publicly available repositories of evidence-based stress management and psychosocial wellness apps going beyond consumer reports (including transparent information on public, private, or government ownership, public launch date, durability, and version history) so that the general public can make informed choices. Potential users should be directed to web-based resources such as PsyberGuide [75] to explore ratings and reviews for digital mental health products [71].

The majority of apps we identified were designed as self-help interventions; they were not necessarily intended for those with psychopathology. For individuals who are interested in seeking self-help via publicly available mHealth interventions, it is advisable to caution them against using this as a replacement to traditional treatment approaches, especially in the case of moderate to severe psychosocial problems. Importantly, the majority of studies found in our review used a sample of convenience (college students and users who have already downloaded the app and are therefore motivated to use them). It is unclear whether these apps would perform similarly if participants had more severe psychopathology symptoms.

Similarly, the role of health care providers and psychosocial clinicians in the mHealth space for an eclectic group of self-help seekers warrants exploration. E-therapist support is infrequently built into consumer apps (11/1009, 1.09%) of apps in our review). In addition to being resource intensive, this level of intervention may not be universally appealing or therapeutically indicated for generally healthy individuals interested in psychosocial self-care. On the other hand, in a clinical population with a serious mental health condition (PTSD), 1 of the studies included in our review found that clinician-supported app use outperformed self-directed app use [40]. This is consistent with previous literature regarding the therapist-patient relationship as a significant predictor of success in psychosocial treatment [76], and it remains to be determined whether app efficacy or engagement could be enhanced when paired with some form of clinician support. Future research should explore the optimal balance between clinician assistance and self-direction in mHealth and for which target audience (mental health support vs mental illness treatment support). At the very least, clinicians working with patients with diagnosed mental illnesses may choose to recommend specific evidence-based apps for skills practice and as a supplement to in-person therapy.

Recently, the Food and Drug Administration has released a timely policy report of its intent to provide regulatory oversight of mobile medical apps “for diagnosis of disease or other conditions, or the cure, mitigation, treatment, or prevention of a disease” and to “apply this oversight authority only to those software apps whose functionality could pose a risk to a patient’s safety if the software apps were not to function as intended” [38]. Exceptions to oversight regulations are made for licensed professionals who create an app solely for use in their own practice or the manufacturing of mobile medical apps solely for use in research, teaching, and analysis and not for commercial use. This may influence the target audience for whom publicly available apps are developed as well as what is available for free. As the majority of apps identified in this review were advertised to consumers under a broad wellness and self-care umbrella, there are important gaps in addressing the unique psychosocial needs of vulnerable groups such as individuals with psychological disorders, individuals with chronic illnesses, and youths. Another important area of research is to explore how users engage with digital health technologies including the leveraging of *big data* analytics; identifying factors that can enhance engagement and usability will help inform the design and optimization of apps for long-term appeal and sustainability [5]. These factors have not yet been explored.

### Limitations

There is no established *gold standard* for searching, evaluating, or reviewing digital health technologies. Recognizing that a methodology for using mobile app search engines to identify apps for review was nontraditional, we leveraged prior research on consumer apps to create a study-specific template and decision rules [17-19]. We relied on product pages on the Apple Store and Google Play to extract data on intervention characteristics, and specific search terms for our literature review which is not without its limitations. Although we provided transparency of our methods here, we recognized that they may not be reproducible. Similarly, it was impossible to construct a *stable, final* database of apps, given the quickly evolving mHealth landscape; new apps are developed, and old apps retired at a rapid rate. In the time frame in which our app store search was conducted, for example, 10.15% (114/1123) apps that were originally included in our database were no longer available 3 months later. Hence, our findings may lack the stability of classical systematic reviews. Similarly, it is possible that app names may have changed from the research design and testing phase reported in peer review publications to its official launch on the Apple Store or Google Play, or that other existing peer-reviewed publications may not have been identified utilizing our search strategy. For example, Happify's website lists two additional published efficacy studies that were not identified by our literature review search terms [77,78]. We were unable to conduct a meta-analysis of RCTs because of the differences in measures and measurement timepoints implemented across studies and relatively few trials with active comparison conditions.

Next, the apps we selected may not be representative of all mHealth programs. We included only free apps or free apps with in-app purchases. It is possible that paid apps significantly differ from free apps with respect to content and efficacy. Furthermore, some in-app purchases included access to an e-therapist, suggesting that secure services require greater monetary resources. As a result, access to effective digital health technologies may represent an unappreciated and important health disparity. However, it is unclear whether and the extent to which the involvement of an e-therapist bolsters outcomes and whether the benefits outweigh the costs; only 1.09% (11/1009) of apps included an e-therapist feature. In addition, the apps included in our review were limited to the English language. This may lead to a Western cultural bias in overrepresentation of apps with active coping strategies that reflect individualistic values and a personal sense of control over stressors [79-81].

### Conclusions

In merging traditional systematic review methodologies with a direct-to-consumer selection criteria for mHealth apps, our study findings suggest that few publicly available stress management and psychosocial wellness apps that are discoverable to self-help seekers are evidence based. Additional research is needed regarding the relative role and utility of mHealth in individual self-care and the care of persons with serious mental illness, the value of mHealth-clinician collaborations, access to mHealth for patients with different resources, and the relative durability of mHealth impact. Meanwhile, clinicians, investigators, and consumers may use findings from our systematic review to navigate the volume of emerging digital health interventions for stress management and wellness.

## Appendix

Multimedia Appendix 1 Search strategies protocol.

Multimedia Appendix 2 Table of intervention components of n=21 stress management apps with published research.

Multimedia Appendix 3 Table of general information for n=21 stress management apps with published research.

Multimedia Appendix 4 Table of findings from efficacy studies (n=25).

Multimedia Appendix 5 Table of usability or feasibility studies (n=10).

## Abbreviations

e-therapist: electronic therapist
mHealth: mobile health
PTSD: posttraumatic stress disorder
RCT: randomized controlled trial
