# Supplementary material for: Android and iPhone Mobile Apps for Psychosocial Wellness and Stress Management: Systematic Search in App Stores and Literature Review
Source: JMIR Mhealth Uhealth. 2020 May 22;8(5):e17798. doi: 10.2196/17798 (PMC7275252; doi:10.2196/17798)
Supplement: Multimedia Appendix 1 [file mhealth_v8i5e17798_app1.docx]

***Multimedia Appendix 1.*** Search Strategies Protocol

**Searching the Apple Store and Google Play: Step 1**

*Inclusion Criteria:* stress management and/or psychosocial wellness focus, available in English, and free for download (including those with free downloads for “basic” subscriptions)

1. We created a list of conventional self-help seeking search terms from mental health and positive psychology background literature, and refined the list based on group consensus within our interdisciplinary research team.
2. We entered into Apple Store and Google Play search engines the final list of 14 conventional self-help seeking search terms:
   1. stress
   2. resilience
   3. goal-setting
   4. relaxation
   5. mindfulness
   6. mood
   7. coping
   8. gratitude
   9. optimism
   10. hope
   11. happiness
   12. sadness
   13. self-compassion
   14. self-care
3. The first 100 apps populated per search term were screened for inclusion.
4. Additionally, we screened the popularity lists embedded in the Apple Store and Google Play in the categories of Health & Fitness and Kids & Family for apps that met inclusion criteria.
5. Duplicates were removed.
6. For each application that met inclusion criteria, two authors independently reviewed and extracted information of interest on intervention content from Apple Store and Google Play product pages.
7. During consensus conversations, our process was to repeat the search to identify the app and leverage product page data to resolve discrepancies.

**Literature Review: Step 2**

*Inclusion Criteria:* research papers published in peer-reviewed journals identified using search terms “[app name]” AND smartphone, available in English, qualitative and/or quantitative studies with original data collection.

*Exclusion Criteria:* conference presentations, editorials, commentaries, and study protocols

1. We then conducted a literature review via Google Scholar, Medline, and PsycINFO databases using the search terms “[app name]” AND smartphone in search of peer-reviewed articles in support of each of the identified applications in Step 1.
2. Two authors retrieved and independently reviewed the full text of all eligible studies to extract outcomes of interest.
3. Two authors independently assessed the quality of included studies using the Cochrane Collaboration’s tool for assessing risk of bias.
4. During consensus conversations, we referred back to the journal articles to resolve discrepancies.
