# Supplementary material for: Android and iPhone Mobile Apps for Psychosocial Wellness and Stress Management: Systematic Search in App Stores and Literature Review
Source: JMIR Mhealth Uhealth. 2020 May 22;8(5):e17798. doi: 10.2196/17798 (PMC7275252; doi:10.2196/17798)
Supplement: Multimedia Appendix 2 [file mhealth_v8i5e17798_app2.docx]

***Multimedia Appendix 2.*** Table of intervention components of n=21 stress management apps with published research.

| App Name | Intervention Components^a^ | | | | | | | | | | | | | | | | | |
| --- | --- | --- | --- | --- | --- | --- | --- | --- | --- | --- | --- | --- | --- | --- | --- | --- | --- | --- |
|  | CBT | Positive Psychology | Mindfulness or Meditation | Resilience | Gratitude^b^ | Relaxation | Planning or  Goal Setting | Educational Materials^c^ | Emotional or  Inspirational Quotes | Journaling | Mood or  Symptom Monitoring | Gamification | Robot or AI Therapist | E-Therapist | Sleep | Hypnosis | Music and Sounds | Peer Supportt and Social Media |
| 10% Happier | ✓ | ✓ | ✓ | X | X | X | X | ✓ | X | X | X | X | X | X | ✓ | X | X | X |
| AEON Mindfulness App | X | X | ✓ | X | X | X | X | X | X | X | X | X | X | X | X | X | X | X |
| Calm^c^ | X | X | ✓ | X | X | X | X | X | X | X | X | X | X | X | ✓ | X | X | X |
| DeStressify | X | X | ✓ | X | X | ✓ | X | X | X | X | ✓ | X | X | X | X | X | X | X |
| Habitica | X | X | X | X | X | X | ✓ | X | X | X | X | X | X | X | X | X | X | X |
| Happify | X | ✓ | ✓ | X | ✓ | X | X | X | X | X | ✓ | X | X | X | X | X | X | X |
| Headspace^c^ | X | X | ✓ | X | X | X | X | X | X | X | X | X | X | X | X | X | X | X |
| JOOL | X | X | X | X | X | X | X | X | X | X | ✓ | X | X | X | X | X | X | X |
| MindSurf | X | ✓ | ✓ | X | X | X | ✓ | X | X | ✓ | X | X | X | X | X | X | X | X |
| MoodMission | ✓ | ✓ | ✓ | X | ✓ | ✓ | X | X | ✓ | X | X | ✓ | X | X | X | X | X | X |
| One Moment Meditation | X | X | ✓ | X | X | X | X | X | X | X | X | X | X | X | X | X | X | X |
| Pacifica | ✓ | ✓ | ✓ | X | X | X | X | X | X | ✓ | ✓ | X | X | X | X | X | X | ✓ |
| Provider Resilience | X | X | X | ✓ | X | X | X | X | X | X | ✓ | X | X | X | X | X | X | X |
| PTSD Coach | ✓ | X | X | X | X | ✓ | X | ✓ | X | X | ✓ | X | X | X | X | X | X | X |
| Smiling Mind | X | X | ✓ | X | X | X | X | X | X | X | ✓ | X | X | X | X | X | X | X |
| Stop, Breathe & Think | X | X | ✓ | X | X | X | X | X | X | X | X | X | X | X | X | X | X | X |
| SuperBetter | X | ✓ | X | X | X | X | X | X | X | X | X | X | X | X | X | X | X | X |
| T2 Mood Tracker | X | X | X | X | X | X | X | X | X | X | ✓ | X | X | X | X | X | X | X |
| Virtual Hope Box | X | ✓ | ✓ | X | X | ✓ | X | X | X | X | X | X | X | X | X | X | X | X |
| Wildflowers Mindfulness | X | X | ✓ | X | X | X | X | X | X | X | ✓ | ✓ | X | X | X | X | X | X |
| Woebot | ✓ | X | X | X | X | X | X | X | X | X | ✓ | X | ✓ | X | X | X | X | X |

^a^ Intervention components were displayed in the table if at least one of the evidence-based apps included said component.

^b^ Gratitude was self-described by app makers and also included "what are you thankful for?" and "three good things" journaling about positive aspects of one's day.

^c^ Educational material was defined as content libraries using written text, audio, and/or videos for psychoeducation.

^d^ ✓:app includes intervention component. **X**: app does not include intervention component.
