# Supplementary material for: Android and iPhone Mobile Apps for Psychosocial Wellness and Stress Management: Systematic Search in App Stores and Literature Review
Source: JMIR Mhealth Uhealth. 2020 May 22;8(5):e17798. doi: 10.2196/17798 (PMC7275252; doi:10.2196/17798)
Supplement: Multimedia Appendix 3 [file mhealth_v8i5e17798_app3.docx]

***Multimedia Appendix 3.*** Table of general information for n=21 stress management apps with published research.

| **App Name** | **App Developer** | **# of**  **Peer-Reviewed Articles** | **Available on:** | | **Average Satisfaction Rating^a^** | **Number of Ratings^b^** | **Cost/Paid Features^c^** |
| --- | --- | --- | --- | --- | --- | --- | --- |
|  |  |  | **Apple Store** | **Google Play** |  |  |  |
| 10% Happier | 10% Happier Inc. | 1 | **✓** | **✓** | 4.8 | 44,879 | Free (Premium: $7.99/month) |
| AEON Mindfulness App | mindfulife | 1 | **✓** | **✓** | 4.0 | 46 | Free |
| Calm | Calm.com, Inc. | 2 | **✓** | **✓** | 4.7 | 604,958 | Free (Premium: $14.99/month) |
| DeStressify | Stress Refuge, Inc. | 1 | **✓** | **✓** | 4.0 | 125 | Free (Paid version: $5.99) |
| Habitica | HabitRPG, Inc. | 1 | **✓** | **✓** | 4.3 | 14,267 | Free |
| Happify | Happify, Inc. | 1 | **✓** | **✓** | 4.0 | 3,614 | Free (Premium: $11.67/month) |
| Headspace | Headspace, Inc. | 8 | **✓** | **✓** | 4.8 | 629,172 | Free (Premium: $12.99/month) |
| JOOL | JOOL Health | 2 | **✓** | **✓** | 4.3 | 12 | Free |
| MindSurf | Tim Carey | 1 | **✓** | **✓** | 3.6 | 9 | Free |
| MoodMission | MoodMission Pty Ltd | 2 | **✓** | **✓** | 3.9 | 180 | Free |
| One Moment Meditation | The One Moment Company LLC | 1 | **✓** | **✓** | 4.7 | 51 | Free |
| Pacifica | Pacifica Labs Inc. | 1 | **✓** | **✓** | 4.6 | 17,346 | Free (Premium: $8.99/month) |
| Provider Resilience | T2 | 1 | **✓** | **✓** | 3.8 | 36 | Free |
| PTSD Coach | US Department of Veterans Affairs | 4 | **✓** | **✓** | 4.3 | 205 | Free |
| Smiling Mind | Smiling Mind | 1 | **✓** | **✓** | 3.9 | 2,193 | Free |
| Stop, Breathe & Think | Stop, Breathe & Think | 1 | **✓** | **✓** | 4.7 | 28,575 | Free (Premium: $9.99/month) |
| SuperBetter | SuperBetter, LLC | 1 | **✓** | **✓** | 4.4 | 9,777 | Free |
| T2 Mood Tracker | National Center for Telehealth & Technology | 1 | **✓** | **✓** | 4.0 | 6,109 | Free |
| Virtual Hope Box | National Center for Telehealth & Technology | 2 | **✓** | **X** | 4.1 | 222 | Free |
| Wildflowers Mindfulness | MobioInteractive | 1 | **✓** | **✓** | 4.5 | 1,211 | Free (Premium: $9.99/month) |
| Woebot | Woebot Labs | 1 | **✓** | **✓** | 4.6 | 5,349 | Free |

^a^Calculated across Apple Store and Google Play = rating / total # of raters where applicable.

^b^Total number of ratings across platforms as of July 2019.

^c^All apps are free to download (includes basic features).

^d^ ✓: app available on platform; X: app not available on platform
