# Supplementary material for: Android and iPhone Mobile Apps for Psychosocial Wellness and Stress Management: Systematic Search in App Stores and Literature Review
Source: JMIR Mhealth Uhealth. 2020 May 22;8(5):e17798. doi: 10.2196/17798 (PMC7275252; doi:10.2196/17798)
Supplement: Multimedia Appendix 4 [file mhealth_v8i5e17798_app4.docx]

***Multimedia Appendix 4.*** Table of findings from efficacy studies (n = 25).

| **App Name** | **Participants** | **RCT** | **Treatment Condition(s)** | **Positive Results** | **Null or Inconclusive Results** |
| --- | --- | --- | --- | --- | --- |
| 10% Happier & Calm[^59^](#_ENREF_59) ^a^ | Adult cancer patients  (N= 128) | **✓** | *Intervention 1 & 2:* 4 weeks of 10% Happier followed by 4 weeks of Calm, or reverse order each for 10 minutes daily^b^  *Intervention 3 & 4*: 4 weeks educational control app followed by 4 weeks of 10% Happier or Calm each for 10 minutes daily | Improved anxiety, depression, sleep disturbance, total symptom burden, fatigue, and physical health for both apps. | No effect on pain intensity, sexual function or quality of life for either app. |
| AEON Mindfulness App[^56^](#_ENREF_56) | Adult app users  (N = 136) | **X** | *Interventio*n: 4 weeks of self-directed use^b^  *Control:* N/A | Improved decentering (i.e., mindfulness skills learning) for naive meditators. | No effect on decentering for experienced meditators. |
| Calm[^57^](#_ENREF_57) | College students  (N = 88) | **✓** | *Intervention*: 10 minutes daily for 8 weeks^b^  *Control:* waitlist | Improved stress, mindfulness, and self-compassion. |  |
| DeStressify^[58](#_ENREF_58)^ | College students  (N = 163) | **✓** | *Intervention*: 5x/week for 4 weeks (paid version)  *Control*: waitlist | Improved trait anxiety (η_p_^2^ = .05) and quality of life (η_p_^2^ = .10). | No effect on stress (η_p_^2^ = .02), state anxiety (η_p_^2^ = .01), sleep quality (η_p_^2^ = .02), work productivity (η_p_^2^ = .02), or depression (η_p_^2^ = .02). |
| Happify^[55](#_ENREF_55)^ | Adult app users  (N = 1,051) | **✓** | *Intervention*: 2-3x/week for 8 weeks^b^  *Control*: Psychoeducation program (reading material with no activities) | Improved resilience and depression and anxiety symptoms. |  |
| Headspace | Adult novice meditators[^54^](#_ENREF_54)  (N = 74) | **✓** | *Intervention*: daily use for 4 weeks^b^  *Control*: waitlist | Improved satisfaction with life, stress, and resilience. |  |
|  | Adult novice meditators[^53^](#_ENREF_53)  (N = 69) | **✓** | *Intervention*: 10 sessions in 4 weeks  *Control*: mindfulness/meditation psychoeducational audiobook | Improved irritability (*d* = 0.44), affect (*d* = 0.47), and stress from external issues (*d* = 0.45). | No difference between groups on stress from internal pressure (*d* = 0.26). |
|  | Adult app users[^52^](#_ENREF_52)  (N = 121) | **✓** | *Intervention:* 10 minutes daily for 10 days  *Control:* list-making app use (Catch Notes) 10 min/day for 10 days | Improved positive affect and depressive symptoms. | No difference between groups on satisfaction with life, flourishing, or negative affect. |
|  | College students[^51^](#_ENREF_51)  (N = 91) | **✓** | *Intervention:* 30 mindfulness meditation sessions over 6 weeks^b^  *Control:* Same timeline, but sham meditations |  | No difference between groups in mindful disposition, critical thinking, or executive functioning. |
|  | Hospital resident physicians[^45^](#_ENREF_45)  (N = 30) | **X** | *Intervention:* Self-guided app usage^b^  *Control:* N/A | Improved mindfulness (*d* = 0.77). | No change over time in positive affect (*d* = 0.38) or negative affect (*d* = −0.15). |
|  | Pediatric nurses  (N = 95)[^49^](#_ENREF_49) | **✓** | *Intervention:* Used app 1x week for 4 weeks in residency classroom^b^ (completed first 10 sessions & were encouraged to use app on their own time)  *Control:* traditionally delivered mindfulness group (in person) 1x per week for 4 weeks | Improved mindfulness. | Showed non-significant improvements in “non-reactivity to inner experience,” compassion satisfaction, and burnout |
|  | Adult cancer patients (N= 19) & their caregivers (N= 9)[^48^](#_ENREF_48) | **X** | *Intervention:* Headspace daily use for 8 weeks  *Control:* N/A | Improved distress and quality of life. | No effect on anxiety, sleep, or fatigue. |
| Headspace & Smiling Mind[^46^](#_ENREF_46) | College students  (N = 208) | **✓** | *Intervention Arm 1*: Headspace daily use for 10 days^b^  *Intervention Arm 2:* Smiling Mind daily use for 10 days  *Control*: Evernote app use | Improved depressive symptoms and college adjustment for both apps. Improved mindfulness for Headspace users. Improved resilience for Smiling Mind users.  Participants who continued to use intervention condition apps after 10-day intervention maintained improvements. | No effect on mindfulness for Smiling Mind users. No effect on resilience for Headspace users. |
| MoodMission^[45](#_ENREF_45)^ | Adults recruited on the Web (N = 226) | **✓** | *Intervention:* Given access to one of three apps—MoodMission, MoodKit or MoodPrism  *Control:* waitlist | Improved depression and coping self-efficacy. | No difference between groups on anxiety, emotional self-awareness, or mental health literacy. |
| Pacifica[^44^](#_ENREF_44) | Adult app users with mild-to-moderate depression or anxiety (N= 500) | **✓** | *Intervention:* 1 month of self-directed app use  *Control:* waitlist | Improved depression, anxiety, stress and self-efficacy. |  |
| Provider Resilience[^43^](#_ENREF_43) | Mental health care providers  (N = 30) | **X** | *Intervention:* Instructed to use app “regularly” over 30 days, but self-directed/not monitored  *Control:* N/A | Improved burnout and compassion fatigue. | No difference between groups in symptom distress, interpersonal relations, social role, or resilience. No change over time in compassion satisfaction. |
| PTSD Coach | Adults with PTSD  (N = 120)[^42^](#_ENREF_42) | **✓** | *Intervention:* 3 months of self-directed app use  *Control:* waitlist |  | No differences in PTSD symptoms, depression, and psychosocial functioning between groups. |
|  | Adults with PTSD  (N = 49)[^41^](#_ENREF_41) | **✓** | *Intervention:* 1 month of self-directed app use  *Control:* waitlist |  | No difference in PTSD symptoms between groups (*d* = −0.25 to -0.33). |
|  | College students  (N = 20)[^40^](#_ENREF_40) | **✓** | *Intervention:* Clinician supported app use (four 20-minute sessions) for 8 weeks  *Control:* Self-directed app use for 8 weeks | Improved PTSD symptoms in both groups. Effect stronger for clinician-supported app use group. |  |
|  | First-time app users (N = 153,834); Repeat users (n = 12,499); user reviews (n = 156)[^39^](#_ENREF_39) | **X** | *Intervention:* Single session of self-directed app use  *Control*: N/A | Improved momentary distress. |  |
| Stop, Breathe & Think[^38^](#_ENREF_38) | Adult app users (N = 13,393) | **X** | *Intervention:* ≥10 app uses over 6 months  *Control*: N/A | Improved mood, anxiety, and depression. |  |
| SuperBetter^[37](#_ENREF_37)^ | Adult app users  (N = 283) | **✓** | *Intervention Arm 1:* Depression-specific SuperBetter app use 10 minutes daily for 1 month  *Intervention Arm 2:* Standard app usage 10 minutes daily for 1 month  *Control:* waitlist | Improved depression, anxiety, life satisfaction, social support and self-efficacy for both app groups. |  |
| Virtual Hope Box[^36^](#_ENREF_36) | Veterans in mental health treatment exhibiting suicidality  (N = 118) | **✓** | *Intervention:* Self-guided app use for 12 weeks + treatment as usual  *Control:* Educational materials about coping with suicidal thoughts + treatment as usual | Improved coping self-efficacy. | No difference between groups on suicidal ideation or perceived reasons for living. |
| Wildflowers Mindfulness[^35^](#_ENREF_35) | College students  (N = 86) | **✓** | *Intervention:* App usage for 10 min/day for 3 weeks  *Control:* Same time frame, but cognitive training app 2048 | Improved mood and stress when symptom monitoring directly after single session of app use.  Improved stress (intervention condition only) and mindfulness skills learning (attentional control, awareness and acceptance) (both conditions) after 3-week intervention period. | No change in attentional control when symptom monitoring directly after app use.  No change in mood after 3-week intervention period. |
| Woebot^[34](#_ENREF_34)^ | College students  (N = 70) | **✓** | *Intervention:* 2 weeks of app usage (up to 20 sessions of AI)  *Control:* E-book information-only control group | Improved depression symptoms. Improved anxiety symptoms in both groups. | No difference in positive or negative affect between groups. |

^a^The 10% Happier & Calm[^59^](#_ENREF_59) and PTSD Coach[^39^](#_ENREF_39) papers are included in both Table 1 and 2 because they evaluated both feasibility and preliminary efficacy.

^b^Versions of both apps tested included premium features.

^c^ ✓: study was an RCT; X: study was not an RCT.

^d^ η_p_^2^ = eta-squared; *d* = Cohen’s *d*. Both statistics are measures of effect size.
