# Supplementary material for: Android and iPhone Mobile Apps for Psychosocial Wellness and Stress Management: Systematic Search in App Stores and Literature Review
Source: JMIR Mhealth Uhealth. 2020 May 22;8(5):e17798. doi: 10.2196/17798 (PMC7275252; doi:10.2196/17798)
Supplement: Multimedia Appendix 5 [file mhealth_v8i5e17798_app5.docx]

***Multimedia Appendix 5.*** Table of usability or feasibility studies (n = 10).

| **App Name** | **Acceptability?** | **Participants** | **Results** |
| --- | --- | --- | --- |
| 10% Happier & Calm^a,^[^59^](#_ENREF_59) | **Mixed** | Adult cancer patients (N = 128) | For 10% Happier and Calm, participants enjoyed app, were satisfied with content, and would recommend to others. A greater proportion of Calm users reported satisfaction on all outcomes. |
| Habitica^[60](#_ENREF_60)^ | **X** | Study 1: Case study (N = 1)  Study 2: College students (N = 45) | Gamification in app found to be counterproductive for users. |
| JOOL[^64^](#_ENREF_64) | **X** | Adult app users (N = 1255) | Push notifications make users more likely to engage with app on weekends. Effect not significant on weekdays. |
| MindSurf^[63](#_ENREF_63)^ | **✓** | College students & staff (N = 23) | Participants found app accessible and the format/style of questioning acceptable. |
| MoodMission^[65](#_ENREF_65)^ | **✓** | Adult beta testers & app users (N = 44) | App rated higher than average on the Mobile App Rating Scale (compared to other health apps on the market). |
| One Moment Meditation[^61^](#_ENREF_61) | **✓** | Hospital care providers (N = 50) | Participants reported that app was acceptable. |
| PTSD Coach | **✓** | Adults with PTSD (N = 45)[^66^](#_ENREF_66) | Participants satisfied with app and found it to be helpful. |
|  | **✓** | First-time app users (N = 153,834); Repeat users (n = 12,499); user reviews (n = 156)[^39^](#_ENREF_39) | Aggregate of mobile analytics for adult app users who downloaded PTSD Coach since its inception demonstrate consistent and strong use over time. User reviews indicate they are satisfied with the app and find it to be helpful. |
| T2 Mood Tracker[^62^](#_ENREF_62) | **✓** | Redeployed soldiers with behavioral health issues (N = 8) | Participants said app was easy to use, useful and beneficial. Participants would recommend app to others and use app in the future. |
| Virtual Hope Box[^67^](#_ENREF_67) | **✓** | Veterans with psychological disorders  (N = 18) | App used more often than conventional intervention. Participants reported that app was easy to use, useful, beneficial and that they would recommend it to others. |

^a^The 10% Happier & Calm[^59^](#_ENREF_59) and PTSD Coach[^39^](#_ENREF_39) papers are included in both Table 1 and 2 because they evaluated both feasibility and preliminary efficacy.

^b^ ✓: study demonstrated acceptability; X: study did not demonstrate acceptability.

^c^PTSD: posttraumatic stress disorder.
